# The Role of Advanced Glycation End Products and Its Soluble Receptor in Kidney Diseases

**DOI:** 10.3390/ijms23073439

**Published:** 2022-03-22

**Authors:** Mieke Steenbeke, Reinhart Speeckaert, Stéphanie Desmedt, Griet Glorieux, Joris R. Delanghe, Marijn M. Speeckaert

**Affiliations:** 1 Nephrology Unit, Department of Internal Medicine and Pediatrics, Ghent University Hospital, 9000 Ghent, Belgium; mieke.steenbeke@uzgent.be (M.S.); stephanie_desmedt@hotmail.com (S.D.); griet.glorieux@ugent.be (G.G.); 2 Department of Dermatology, Ghent University Hospital, 9000 Ghent, Belgium; reinhart.speeckaert@ugent.be; 3 Research Foundation Flanders, 1000 Brussels, Belgium; 4 Department of Diagnostic Sciences, Ghent University, 9000 Ghent, Belgium; joris.delanghe@ugent.be

**Keywords:** advanced glycation end products (AGEs), chronic kidney disease (CKD), soluble receptor for AGEs (sRAGE)

## Abstract

Patients with chronic kidney disease (CKD) are more prone to oxidative stress and chronic inflammation, which may lead to an increase in the synthesis of advanced glycation end products (AGEs). Because AGEs are mostly removed by healthy kidneys, AGE accumulation is a result of both increased production and decreased kidney clearance. On the other hand, AGEs may potentially hasten decreasing kidney function in CKD patients, and are independently related to all-cause mortality. They are one of the non-traditional risk factors that play a significant role in the underlying processes that lead to excessive cardiovascular disease in CKD patients. When AGEs interact with their cell-bound receptor (RAGE), cell dysfunction is initiated by activating nuclear factor kappa-B (NF-κB), increasing the production and release of inflammatory cytokines. Alterations in the AGE-RAGE system have been related to the development of several chronic kidney diseases. Soluble RAGE (sRAGE) is a decoy receptor that suppresses membrane-bound RAGE activation and AGE-RAGE-related toxicity. sRAGE, and more specifically, the AGE/sRAGE ratio, may be promising tools for predicting the prognosis of kidney diseases. In the present review, we discuss the potential role of AGEs and sRAGE as biomarkers in different kidney pathologies.

## 1. Introduction

Advanced glycation end products (AGEs) are a diverse set of compounds formed from the non-enzymatic interaction of reducing sugars and associated metabolites with proteins and amino acids, also known as the Maillard reaction [1]. Protein glycation is caused by a nucleophilic addition reaction between a free amino group from a protein and a carbonyl group from a reducing sugar, which leads to the production of an unstable, freely reversible Schiff base. In the presence of a transition metal, this base is rearranged to a more stable intermediate, an Amadori product, which is then oxidized to generate the final AGE [2]. AGEs can also be generated as a result of glucose autoxidation and oxidative stress. A detailed overview of the complex and heterogeneous pathways of chemical formation of AGEs, encompassing several precursors and processes, has previously been published [3]. N^6^-carboxymethyl-l-lysine (CML), N^6^-carboxyethyl-l-lysine (CEL), S-carboxymethyl-l-cysteine (CMC), S-(1-carboxyethyl)-l-cysteine (CEC), methylglyoxal-derived hydroimidazolone (MG-H1), furosine, and pentosidine are the most routinely detected AGEs [1,4,5]. Intracellular proteolysis of AGE-modified proteins results in the formation of AGE-free adducts that are discharged into the circulation and can contribute to systemic consequences [6]. AGEs have a role in the detrimental legacy effect of long-term inadequate glycemic management on microvascular diabetic complications in people with diabetes mellitus (DM) [7,8].

**Figure 1:**
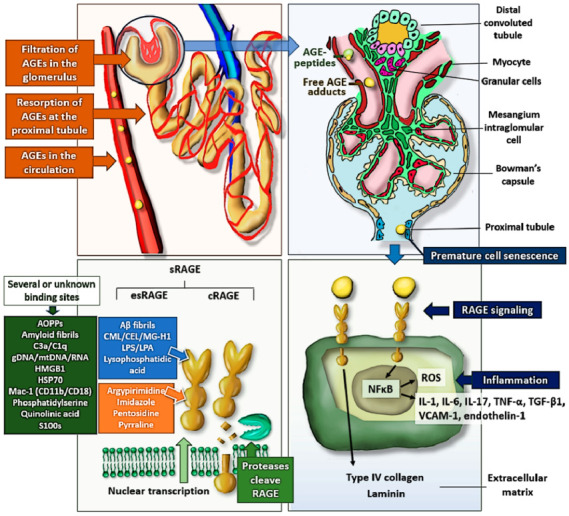
At the side of the kidney, circulating AGEs are raised by both increased generation and poor renal clearance in illnesses associated with high levels of inflammation and oxidative stress. Following glomerular filtration of the AGEs, some of the AGE-free adducts are actively reabsorbed and secreted by the proximal tubular cells. When AGEs connect with their cell-bound receptors in the renal interstitium and mesangium, they can activate the p21 protein, Janus kinase 1 and 2, and NADPH oxidase. The activation of transcription factors, such as NF-κB and ISRE, leads to the synthesis of proinflammatory cytokines, such as TNF-α, interleukins, and VCAM-1, which contribute to inflammation and endothelial dysfunction, leading to the progression of chronic illness. In addition to the membrane-bound form, the soluble form of RAGE (sRAGE) has been found, which is composed of two forms: cRAGE and esRAGE. HMGB1, S100/calgranulin, and β-amyloid protein are all RAGE ligands that may bind to sRAGE. By functioning as a decoy for AGEs and other RAGE ligands without initiating the signaling cascade, sRAGE is intended to mitigate the deleterious consequences of AGE-RAGE complex activation. Abbreviations: AGE: advanced glycation end product; AOPPS; advanced oxidation protein products; CEL: N6-carboxyethyl-l-lysine; CML: N6-carboxymethyl-l-lysine; cRAGE: cleaved RAGE; esRAGE: endogenous secretory RAGE; HMGB1: high-mobility group box 1; HSP: heat shock protein; IL: interleukin; LPA: lipopolysaccharide lipid component A; LPS: lipopolysaccharide; Mac-1: macrophage-1 antigen; MG-H1: methylglyoxal-derived hydroimidazolone; NF-κB: nuclear factor-kappa B; RAGE: receptor for AGEs; ROS: reactive oxygen species; sRAGE: soluble RAGE; TGF-β1: transforming growth factor-beta 1; TNF-α: tumor necrosis factor-alpha; VCAM-1: vascular cell adhesion molecule-1.

An increasing body of research indicates that AGEs may have a role in the development of a variety of illnesses. AGEs have a role in the development of diabetic, vascular, and uremic problems [9,10,11]. Although the precise mechanisms of action of AGEs are unknown, they are most likely the outcome of the interaction with their membrane-bound receptor (RAGE) [12,13,14]. The AGE-RAGE complex (Figure 1) can specifically activate the p21 protein, stimulating other signaling pathways such as extracellular signal-regulated kinase (ERK), c-Jun N-terminal kinase (JNK), and mitogen-activated protein kinase (MAP), as well as Janus kinase 1 and 2/Signal transducer and activators of transcription (JAK1&2/STAT1), and nicotinamide adenine dinucleotide phosphate (NADPH) oxidase, which generate directly and indirectly reactive oxygen species (ROS) [15]. The activation of transcription factors such as nuclear factor kappa B (NF-kB) and the interferon-sensitive response element (ISRE) results in the production of interleukins, pro-inflammatory cytokines such as tumor necrosis factor-alpha (TNF-α), and vascular cell adhesion molecule-1 (VCAM-1), contributing to inflammation and endothelial dysfunction, leading to the progression of chronic illness [15,16,17,18,19].

RAGE also exists as a soluble molecule called sRAGE, which is composed of two forms: cleaved RAGE (cRAGE), which results from proteolytic cleavage of membrane-bound RAGE, and endogenous secretory RAGE (esRAGE), a splice variant with the extracellular domains but lacking the intracytoplasmic and transmembrane domains [20]. sRAGE is a 48-kDa positively charged cleavage product that maintains the ligand binding site but loses the transmembrane and signaling domains [21,22]. In addition to binding AGEs, sRAGE has attracted attention for its capacity to bind a variety of RAGE ligands, including high-mobility group box 1 (HMGB1), S100/calgranulin, and β-amyloid protein [23]. sRAGE is expected to counteract the negative effects of AGE-RAGE complex activation by acting as a decoy for AGEs and other RAGE ligands without activating the signaling cascade [23,24]. AGEs might also bind to oligosaccharyltransferase-48 (AGE-R1), 80K-H phosphoprotein (AGE-R2), galectin-3 (AGE-R3), macrophage scavenger receptors, lectin-like oxidized low-density lipoprotein receptor-1 (LOX-1), fasciclin, EGF-like, laminin-type EGF-like, and link domain-containing scavenger receptor-1/2 (FEEL1/2) and CD36. It is unknown if a specific form of AGE preferentially binds to a certain type of receptor, or whether there is any tissue-specific interaction between ligand and receptor. AGE-R1, AGR-R3, and macrophage scavenger receptor types I and II are involved in AGE detoxification and degradation [25].

The purpose of this review is to provide an overview of the role of AGEs and sRAGE in diverse kidney disorders. In the kidneys, the glomeruli filter the AGEs, which are then reabsorbed by the renal proximal tubules [26]. Their clearance as well as tubular reabsorption are both difficult and varied [27,28]. AGEs interact with RAGE and other matrix cell receptors, and accumulate in the renal interstitium and mesangium, where they stimulate the production of type IV collagen and laminin in the extracellular matrix and produce irreversible cross-linked protein formations [29,30]. They cause premature cell senescence in the proximal tubule [31]. Elevated circulating AGEs are caused by both increased production in disorders linked with high levels of inflammation and oxidative stress, as well as impaired renal clearance, as in chronic kidney disease (CKD) [32]. In line with the major involvement of the kidneys in the clearance of circulating AGE-free adducts, serum AGE levels are inversely related to the estimated glomerular filtration rate (eGFR) [6,33,34,35,36].

## 2. Analytical Aspects

AGEs may be measured in plasma, urine, or tissues, with skin being the most commonly utilized. The most reliable technology for determining AGE levels is gas chromatography–mass spectrometry (GC-MS) [37]. Gas or liquid chromatography (LC)-MS is sensitive, but not without technical limitations. High-performance liquid chromatography (HPLC) [38], enzyme-linked immunosorbent assay (ELISA) [39], immunohistochemistry [40], and autofluorescence [41] are now the most often employed detection techniques. At this moment, ELISAs lack standardization, whereas skin autofluorescence (SAF) is neither specific nor quantifiable for individual AGEs, and is influenced by skin pigmentation. The components of a mixture formed by the interaction of the used substances and chromatography columns can be analyzed by HPLC, which can identify the amounts of AGEs based on the intensity of their fluorescence. HPLC also enables a quicker investigation of protein-bound AGEs while determining their effect on the biologic properties of tissues [15]. Using MS, chemical compounds may be identified, characterized, and quantified based on fragmentation patterns. GC-MS is a method for detecting the activity of AGEs and assessing oxidative stress markers [42].

## 3. Chronic Kidney Disease

Patients with CKD have malnutrition; accelerated biological aging; cardiometabolic, musculoskeletal, and cerebral problems; functional and quality-of-life deficits; and an increased mortality rate. Excessive oxidative stress, chronic inflammation, and uremia may all lead to an increase in AGE formation, which promotes CKD morbidity and death [43]. Serum AGE concentrations are inversely related to residual kidney function and may rise up to 5 to 100-fold in patients with end-stage kidney disease (ESKD) [44]. The reduced excretion mechanism in chronic kidney failure is caused by AGE buildup in the tissue, where they cross-link with collagen, causing vascular stiffness [43]. AGEs contribute to the advancement of kidney disease with putative pathways including binding to the RAGE and generating endothelial dysfunction, oxidative stress, inflammation, and podocyte damage [45]. The potential roles of specific AGEs in the development of kidney diseases are presented in Table 1 [1,2,46,47,48,49,50,51,52,53,54,55]. sRAGE levels are linked to kidney function, AGEs, and inflammation, but they can also be altered genetically. An overview of human studies investigating the role of AGEs and sRAGE in CKD is presented in Table 2 [9,10,11,56,57,58,59,60,61,62,63,64,65,66,67].

In the Women’s Health and Aging Study I, a prospective cross-sectional study of 548 older community-dwelling women (52% with a decreased eGFR of <60 mL/min/1.73 m^2^ and 15% with DM at baseline), increased serum CML and sRAGE were independently linked with lower eGFR and seemed to predict reduced eGFR [56]. At 1 year follow-up, mean plasma AGE levels remained nearly the same in a cohort of 64 older individuals (63% with DM) with a rather steady eGFR (eGFR: 27 ± 10 mL/min/1.73 m^2^), although sRAGE isoforms decreased significantly (*p* < 0.0001). The AGE/sRAGE ratios rose (*p* = 0.004), but variations in AGEs and RAGEs isoforms were not associated with changes in eGFR during follow-up. This absence of relationship implies that plasma AGE concentrations were related to the individual pro-oxidant environment rather than kidney function. The increased AGE/sRAGE ratio at follow-up showed a higher risk of AGE-dependent development of both CKD and other co-morbidities. The potential usefulness of the AGE/sRAGE ratio as a prognostic tool should be examined further [9].

A cross-sectional analysis of the Atherosclerosis Risk in Communities (ARIC) study (*n* = 1874) found that kidney-related variables (eGFR, albuminuria, and CKD risk based on eGFR and albuminuria stages) were linked to increased levels of AGE-CML, sRAGE, and esRAGE. After controlling for basic demographic information and body mass index (BMI), C-reactive protein (CRP), black race, and advanced glycosylation end product-specific receptor (*AGER*) genetic variations (rs2070600 and rs2071288) were highly related to lower serum sRAGE and esRAGE concentrations. Fructosamine and glycated albumin were moderately linked with AGE-CML, indicating that serum AGE-CML may also represent short-term glycation (2–4 weeks). Hemoglobin A1c (HbA1c) was not linked with serum AGE-CML, sRAGE, or esRAGE after adjusting for baseline demographic variables and BMI. In this community-based group, AGE-CML and sRAGE were linked to renal, inflammatory, and genetic markers rather than DM. However, the authors concluded that these biomarkers lack specificity when tested by ELISA and have limited use in assessing the involvement of these compounds in DM [57].

Significantly higher blood concentrations of AGEs, sRAGE, and the AGE/sRAGE ratio were found in ESKD patients (including both hemodialysis (HD) and peritoneal dialysis (PD)), in comparison with healthy individuals [58,65]. Increased AGE synthesis and accumulation in uremia leads to RAGE up-regulation, which has been related to interstitial peritoneal fibrosis and vascular sclerosis [66]. Exposure to high-glucose PD fluids may exacerbate such alterations [67]. The amount of AGEs was non-significantly different in uremic patients with and without DM [68]. Baseline SAF, but not serum CML, was substantially linked with the risk of all-cause and cardiovascular disease (CVD) mortality in the China Cooperative Study on Dialysis (CCSD), a multicenter prospective cohort study of 1634 HD patients. The positive relationship between tissue AGEs and all-cause mortality was more significant in individuals with shorter dialysis vintage or lower CRP levels, indicating that tissue AGE accumulation might be an early predictor of all-cause or cardiovascular (CV) death. The processes by which AGE buildup in tissue raises mortality risk are unknown, although they are biologically probable. The accumulation of AGEs in arterial walls may result in cross-links between structural proteins such as collagen and elastin, leading to arterial stiffness [59].

A negative connection was discovered between sRAGE and the AGE/sRAGE ratio. In HD patients, sRAGE is increased due to decreased kidney function, which is a strong determinant of sRAGE levels, and is not significantly modified by dialysis therapy [60,61,62,63,64,69,70]. Besides the rise in circulating AGEs and sRAGE due to impaired kidney function, the sRAGE concentration might also be elevated in uremia to defend against the harmful effects of AGEs, within a counter-regulatory mechanism against vascular endothelial injury [10,56,63,64,68]. sRAGE showed a negative relationship between residual diuresis and acute-phase reactants (fibrinogen and orosomucoid) [60]. The negative association of sRAGE to inflammation in HD patients suggests that it behaves similarly to the general population, where lower levels were detected in states associated with illness consequences, inflammation, or microinflammation [71,72].

The AGE/sRAGE ratio might be used as a general risk biomarker for ESKD [58]. sRAGE was not only connected with DM and renal disorders, but was also associated with CVD (independent predictors) and their risk factors (hypercholesterolemia), regardless of DM. This underscores the prognostic relevance of AGEs and sRAGE in HD patients, independent of the underlying etiology, in predicting CVD risk [68]. sRAGE influences the vascular calcification process, even after controlling for established CV risk variables [61,73], and has anti-inflammatory properties by inhibiting the interactions of AGEs with cell surfaces [61]. Reduced sRAGE levels may play an important role in systemic inflammation and carotid atherosclerosis in PD patients [73]. AGE-RAGE interaction may stimulate osteoblast-like growth of vascular smooth muscle cells via the RAGE/p38 MAP signaling pathway [74,75]. The A419C (E111A) polymorphism of the *glyoxalase I* gene associates with serum sRAGE levels. The CC variant shows a genetic predisposition to vascular complications in HD patients [76].

A rise in sRAGE levels has been linked with a substantial increase in mortality risk in HD and PD patients [11,61]. The positive correlation with brain natriuretic peptide (BNP) levels implies that sRAGE may have a role as a predictive indicator for mortality, indicating an active process of cardiac remodeling [11]. However, other studies could not confirm the association between sRAGE and peripheral vascular disease, cerebrovascular illness, or mortality [60,62,69].

## 4. Diabetic Nephropathy

Diabetic nephropathy (DN), or diabetic kidney disease (DKD), is a major microvascular complication of DM and the most common cause of ESKD, characterized by the presence of increased urine albumin excretion, diabetic glomerular lesions, and loss of GFR [77]. DKD risk is associated with persistent hyperglycemia [78] and can be lowered in people with type 1 diabetes mellitus (T1DM) and early type 2 diabetes mellitus (T2DM) by maintaining strict glycemic control [79,80]. In contrast, in those with more chronic T2DM, intensive glycemic control had only a minor effect on DKD progression [81], raising the possibility that additional risk factors associated with long-term T2DM that are not easily improved by intensive glycemic control may play a significant role in kidney function deterioration. Although glycated hemoglobin (HbA1c), an Amadori rearrangement product formed by hemoglobin binding to glucose in red blood cells, has been demonstrated to be a reliable prognostic marker in the general diabetic population, it may not be effective in diabetes patients with chronic renal impairment [82]. Table 3 gives an overview of human studies investigating the role of AGEs and sRAGE in DN [6,34,35,36,83,84,85,86,87].

In animal studies, increasing AGE exposure induced microvascular dysfunction and DKD [88,89]. Higher levels of numerous AGE-free adducts predicted poorer morphologic characteristics of DN in people with T1DM or T2DM [34,36]. In individuals with T1DM or T2DM, serum and tissue AGE levels were considerably higher than in non-diabetic controls. When compared to diabetic individuals without renal illness, diabetic patients with ESKD had about double the amount of tissue AGEs. AGEs build at a faster-than-normal rate in diabetic individuals’ arteries and circulation. In DN, the rise in circulating AGE peptides corresponds to the level of renal functional impairment [85]. Human and experimental DN models have consistently indicated the presence of AGE in the glomeruli and tubules, most notably in the glomerular basement membrane, mesangial cells, tubular cells, and podocytes [90]. Chronic hyperglycemia (and associated mechanisms such as oxidative stress) might have a more important role in AGE production than more recent ambient glycemic control. Once generated, AGE-modified proteins are long-lasting, are not easily changed by glucose management, and are prominent risk factors for microvascular complications of diabetes that might be essentially independent of glycemic control [91].

Eleven plasma protein-bound AGEs were detected using LC-MS in 466 individuals in the Diabetes Control and Complications Trial/Epidemiology of Diabetes Interventions and Complications (DCCT/EDIC) study at three timepoints (TPs): DCCT years 4 (TP1) and 8 (TP2), and EDIC years 5/6. (TP3). Methionine sulfoxide (MetSOX) was linked to DKD at TP1 (eGFR < 60 mL/min/1.73 m^2^ and albuminuria). When age, gender, BMI, DM duration, and mean updated HbA1c were all taken into account, a positive relationship between fructose-lysine and CML and albuminuria was discovered. This points to oxidative stress playing a role in the etiology of DKD [84]. CEL and MG-H1 predicted renal function loss (RFL), defined as a 40% drop in measured GFR from baseline, and connected with the severity of DKD lesions in a longitudinal study of 169 Pima Indians with T2DM and early-stage DN (median follow-up of 8 years). When combined with established renal risk variables, MG-H1 greatly enhanced the accuracy of RFL prediction [34]. Declining kidney function, with corresponding decreases in renal clearance of these AGEs [2], was principally responsible for their growing concentrations seen over follow-up, and is why the time-dependent model did not enhance RFL prediction. Some serum AGEs may be effective indicators for progressive DKD and may have a pathophysiological role in its progression [34]. DN is distinguished by increased circulating CML and CML accumulation in tissues. CML might have a role in the development of DN by interfering with the intracellular feedback control of cholesterol. Inhibiting CML-induced lipid accumulation may have renoprotective effects in the development of DN [92].

The Natural History of Diabetic Nephropathy Study [35] evaluated the connection between AGEs and the course of DN in 103 participants with T1DM over a 5-year period. Using a logistic regression model to link each biomarker to the likelihood of a person being classified as a fast progressor, CML, CEL, and MG-H1, but not HbA1c, revealed a significant association to the likelihood of fast progressor. Because two of the three predictive biomarkers are methylglyoxal end products (CEL and MG-H1), these findings imply a role for increased methylglyoxal levels in the development of DN, which is consistent with increased methylglyoxal cellular synthesis in DN progressrs [34]. These AGEs may be early indications of the progression of significant DN damage [35]. The efficacy of a multicomponent AGE panel for predicting RFL when supplemented to regular clinical measures in T2DM has been explored. CEL and CML, as well as 3-deoxyglucosone, MG-H1, and glyoxal hydroimidazolones (G-H1), were measured in baseline blood samples from participants in the Action to Control Cardiovascular Risk in Diabetes (ACCORD) trial (*n* = 1150) and Veterans Affairs Diabetes Trial (VADT) (*n* = 447), respectively. After adjusting for baseline and follow-up HbA1c and other risk variables in the ACCORD trial, the AGE score based on serum levels of the five AGE-free adducts was linked with macroalbuminuria, a decline in eGFR, a sustained 30% decrease in eGFR, a sustained 40% decrease in eGFR, and high-risk CKD (eGFR < 30 mL/min/1.73 m^2^ with urine albumin-to-creatinine ratio (uACR) < 30 mg/g, or eGFR < 45 mL/min/1.73 m^2^ with uACR 30–299 mg/g, or eGFR < 60 mL/min/1.73 m^2^ with uACR ≥ 300 mg/g). A similar relationship between baseline AGE score and eventual renal function impairment in a different T2DM cohort verified the durability of this finding. A predictive model for a sustained 30% decrease in eGFR that included clinical risk factors as well as an AGE score derived from the ACCORD data distinguished between participants who developed this outcome in VADT and those who did not, indicating the potential predictive value of a standard AGE score across T2DM populations. As different pathways from specific precursors lead to individual AGE-free adducts [93,94], the blood concentrations of individual AGEs or their relationships to DM complications may be influenced by a variety of clinical and demographic factors, which may differ between study cohorts. As a result, the composite AGE score may give a more accurate and consistent measure of the total AGE burden, as well as a more accurate predictive estimation of AGE-related hazards across groups. When combined with established risk factors for DKD, a higher AGE score enhanced prediction of whether or not the patients will develop future sustained function loss. These findings add to the evidence that AGEs play a causative role in DN irrespective of glycemic management, and they point to the efficacy of the composite AGE panel in predicting long-term loss in kidney function. The link between AGEs and renal illness is not merely due to reverse causation, with early decreases in GFR increasing AGE-free adduct levels. Even in individuals with normal kidney function (eGFR > 90 mL/min 1.73 m^2^), there was a link between a higher AGE score and a higher likelihood of poor renal outcomes. Because they are related with two separate renal outcomes, the deterioration of both proteinuria and creatinine clearance, plasma AGEs have a wide pathophysiologic relationship with acute kidney problems [6].

Plasma levels of sRAGE were not substantially different among CKD patients with variable glycemic control status in a study of 150 CKD patients and 64 non-CKD patients. Regardless of glycemic control status, plasma sRAGE levels were higher in diabetic individuals with CKD than in those without CKD. There were no interactions between CKD level and glycemic control status in terms of sRAGE. Despite the benefits of excellent glycemic control, well-controlled DM in CKD did not change the activities of enzymatic antioxidants or sRAGE levels, suggesting that this is not the predominant strategy for dealing with oxidative stress. The presence of CKD may negate the impact of glycemic management on enzymatic antioxidant activity and sRAGE levels [85]. However, another cross-sectional study of 76 CKD stage 5 on dialysis (CKD-G5D) patients found higher plasma sRAGE levels in DM CKD-G5D patients compared to non-DM CKD-G5D patients, implying the presence of a glycated milieu in all CKD-G5D patients, independent of the presence of DM. Its subsequent rise in DM might be explained by an increase in sRAGE in DM as a defense system against glycated products. sRAGE was found to be an independent predictor of BNP levels, indicating that it might be used as a measure of cardiac remodeling in CKD-G5D patients. Its elevation may be a possible preventive mechanism against the increased risk of CV problems associated with AGEs and inflammation. Mechanistic investigations are needed to confirm the causal link between sRAGE and CV risk in these patients [86]. Antioxidant supplements and sRAGE treatment can help diabetic CKD patients reduce oxidative stress. However, due to the wide range of potential combinations and confounding factors, it is difficult to evaluate the modulatory effects of pharmaceuticals (antidiabetic drugs, antihypertensive drugs, statins) on antioxidants and sRAGE [85].

A prospective cohort study from the Hong Kong diabetic biobank looked at the relationship between SAF and kidney failure in 3725 people with T2DM who did not have ESKD. During a median follow-up of 1.8 (1.1–3.1) years, 411 patients had incident ESKD or had a 30% decrease in eGFR. After controlling for risk variables such as baseline eGFR and uACR, SAF was linked to kidney disease progression and yearly drop in eGFR. Reduced eGFR (12.9%) and increased uACR (25.8%) accounted for 38.7% of the effect of SAF on renal outcome [87].

## 5. Atherosclerosis

Atherosclerosis is a chronic disease process involving various biological elements such as myocytes, endothelial cells, lymphocytes, and platelets, as well as adhesion molecules and cytokines [95]. This condition is enhanced by the buildup of AGEs caused by aberrant lipoprotein metabolism, the cross-linking of matrix proteins required for endothelial function, and platelet aggregation. CKD patients have higher blood glycated low-density lipoprotein (LDL) concentrations, which are more prone to oxidation and removed from the circulation at a slower pace than non-glycated LDL. Increased oxidation and reduced clearance of AGE-modified LDL may contribute to CKD patients’ increased risk of atherosclerosis [96]. We give an overview of human studies investigating the role of AGEs and sRAGE in atherosclerosis in Table 4 [62,97,98,99,100,101,102,103].

In the ARIC Study, which included 151 CKD patients, 152 ESKD patients, and 1218 healthy people, baseline serum sRAGE levels were inversely associated with baseline eGFR, and positively correlated with other kidney filtration markers (β-trace protein, β_2_ microglobulin). After adjusting for gender, age, and race, one interquartile range higher log_10_-transformed sRAGE was associated with the development of CKD (*p* = 0.02) and ESKD (*p* < 0.001), but not after adjusting for baseline kidney function. These findings show that circulating levels of sRAGE may be directly influenced by a kidney’s inability to filter endogenous chemicals properly, or that sRAGE directly affects kidney function [97]. The mechanism through which sRAGE affects tissues and signaling pathways is not completely understood. One theory is that sRAGE concentrations reflect the level of AGE-RAGE activity, which might lead to negative health implications [12,17,104]. RAGE messenger ribonucleic acid (mRNA) expression rises in the presence of high amounts of circulating AGEs, resulting in up-regulation of RAGE synthesis [98]. Because sRAGE is a cleavage product of RAGE, its plasma concentration might be related to the quantity of RAGE generated. Another possibility is that, like AGEs, higher levels of circulating sRAGE are caused by impaired renal clearance and subsequent sRAGE buildup in those with poor kidney function [99,105]. The kidney’s capacity to filter properly may have a role in determining sRAGE levels in the blood. Future research will be needed to look into the specific pathways that link sRAGE to the risk of kidney disease [97].

In selected atherosclerotic disorders, CKD patients (50 CKD stage 3–4 and 35 CKD-G5D) had substantially higher quantities of AGEs and sRAGE than patients with abdominal aortic aneurysm and aortoiliac occlusive disease. The increase in AGE levels might be explained by increased oxidative stress and a loss of kidney function. The underlying pathophysiological process may be based on growing protein stiffness caused by protein cross-linking, cytokine, and enhanced adhesion molecule expression [100]. AGEs have been detected in the atherosclerotic plaques of patients with CKD [101]. Because of the extra complications involved in the etiology of CKD, the glycation process is more severe in CKD patients. A positive association was found between the level of AGEs and the concentration of sRAGE. AGEs induce the production of sRAGE, showing an increase in the production of scavenging receptors to defend against growing oxidative stress in CKD [100]. The amount of sRAGE in CKD-G5D patients was considerably higher than in CKD stage 3–4 patients, suggesting that it may be a protective mechanism against increasing oxidative stress and inflammation [62]. A significantly higher AGE/sRAGE ratio was also seen in HD patients as compared to those with abdominal aortic aneurysm and aortoiliac occlusive disease. The greater amount of AGEs per sRAGE may indicate ligand-RAGE receptor interaction, which is promoted in CKD patients [100].

In a study of 12 CKD patients (average eGFR of 32 mL/min/1.73 m^2^) and 49 healthy control people [104], there were significant negative correlations between sRAGE and intima–media thickness and plaque number in CKD patients. The slopes of intima–media thickness and plaque number to sRAGE in CKD patients were substantially steeper than in controls. In CKD patients, there was a strong interaction between sRAGE and smoking for predicting atherosclerotic plaques. A high sRAGE may operate as a vasculoprotective factor and counteract the vasculotoxic impact of AGE accumulation. Longitudinal observations and intervention studies are needed to determine if this connection is causative. It is doubtful that measuring sRAGE is beneficial in clinical practice for identifying people at increased risk of atherosclerosis. Strong negative associations between sRAGE and left ventricular mass index (LVMI) and mean wall thickness (MWT) were discovered, which were essentially independent of traditional and non-traditional risk factors [103]. A one-log-unit rise in sRAGE was related to an 82% risk reduction of left ventricular hypertrophy (LVH) in CKD patients. This connection suggests that the RAGE pathway may be a risk factor for LVH, and that blocking the accumulation of connective tissue in the myocardium with the endogenous decoy receptor sRAGE may reduce LVH in the same cohort [106].

## 6. Lupus Nephritis

Lupus nephritis (LN), an inflammation of the kidney that encompasses diverse patterns of kidney disease such as glomerular, tubulointerstitial, and vascular pathology, is a severe complication of systemic lupus erythematosus (SLE) that progresses to ESKD in approximately 10% of patients within 5 years of onset [107,108]. Pentosidine and CML have been found in proliferative glomerular tufts and crescents of active LN patients. AGEs are formed during the acute phase of glomerular damage, prior to glomerular scarring. The development of AGEs by enzymatic oxidative effects on external glomerular matrix proteins may explain this phenomenon [109]. The generation of ROS and proteases (myeloperoxidase) by neutrophils might transform hydroxy-amino acids into glycolaldehyde, a precursor to CML [110].

Plasma sRAGE levels were adversely linked with SLE disease activity index in a Chinese cross-sectional investigation of 27 consecutive female patients. esRAGE and sRAGE plasma concentrations were considerably higher in LN patients without a flare than in those who did have a flare [111]. This lends credence to the idea that sRAGE can operate as a ligand decoy [112]. Because of the higher HMGB1 level in LN patients without flare compared to healthy controls, the plasma concentration of sRAGE was also higher in this group. Nonetheless, no correlation was discovered between sRAGE levels and HMGB1 levels, most likely because additional RAGE ligands (S100 proteins, serum amyloid A) may also be implicated in this RAGE inflammatory axis. Complement C3 levels were inversely associated with the esRAGE/sRAGE ratio. Plasma sRAGE might be used as a biomarker for disease activity and as a future treatment target in SLE. Reduced levels of sRAGE and/or esRAGE may explain ligand RAGE pathway hyperactivity and insufficient endogenous protective response, making them possible therapeutic targets [111]. 

*AGER* polymorphisms have been linked to susceptibility to SLE and LN. When compared to healthy controls, the C allele of *AGER*-429 T/C, the T allele of *AGER*-374 T/A, and the G allele of *AGER*-2184 A/G were considerably more frequent in SLE and LN. During the first two years of therapy, the C allele of *AGER*-429 T/C, the A allele of *AGER*-374 T/A, and the G allele of *AGER*-2184 A/G polymorphism were substantially related to higher proteinuria and lower kidney function in LN. Although *AGER* polymorphisms may have a causal relationship with illness susceptibility in SLE and disease severity in LN, no genotype-sRAGE connection was detected [113].

## 7. Renal Amyloidosis

Renal amyloidosis is an uncommon and difficult-to-treat protein misfolding condition that causes progressive kidney failure [114]. The function of AGE in amyloidogenesis and amyloid-related complications is still being studied. The presence of CML-modified amyloid protein in renal tissues (both in glomeruli and interstitium) of patients with amyloid A (AA) amyloidosis was investigated in 33 kidney biopsy samples from patients with systemic amyloidosis (19 with AA amyloidosis and 14 with amyloid light-chain (AL) amyloidosis). Collagen type IV and laminin were colocalized in AA-amyloid positive regions. Cases with AL-amyloid with a long history of nephropathy revealed strong staining for CML in the glomeruli and interstitium, but no staining for collagen IV or laminin in the amyloid deposits. This suggests that CML may require long incubation to form in AL amyloid protein. Pyrraline, a non-oxidative AGE, was not detected in AA or AL amyloidosis [115]. A case study of 25 patients revealed similar results. AGEs were found in much lower levels in AL or transthyretin (ATTR) amyloidosis than in AA amyloidosis. AGEs in AA and AL amyloid are a diverse group, and CML contributes only partially to AGE formation in AA and AL amyloid. If AGEs or CML have a role in the etiology of AA amyloidosis, it is not through the alteration of serum amyloid A (SAA) or AA fibril proteins. Although the presence of AGEs does not exclude the development of AA amyloid fibrils, they can impact amyloidogenesis by activating RAGE. In all individuals with AA amyloidosis, RAGE was shown to be spatially associated with amyloid deposits. The existence of RAGE, like AGEs, may be connected to the underlying illness. Future research is needed to determine if serum AGE or CML levels can be utilized to predict the severity or duration of a chronic inflammatory condition, and, consequently, the onset or progression of AA amyloidosis [40].

## 8. Acute Kidney Injury

In the majority of patients admitted to the intensive care unit (ICU), oxidative stress plays a significant role in the course and final fate. AGEs accumulation has only been documented in chronic situations. However, it may also develop abruptly as a result of oxidative stress. Oxidative stress results in the development of reactive carbonyl molecules, which then react with protein to generate AGEs [116]. An acute incident may generate immediate oxidative stress in the ICU patient, as well as subsequent AGE buildup, which may cause further oxidative stress, possibly resulting in a vicious cycle with progressive multiple organ failure. In contrast to chronic AGE accumulation, current research has focused on the less well-known acute AGE accumulation. One may speculate that the acute AGE buildup is damaging due to intracellular ROS production and depletion of antioxidant systems [18,117]. The binding of AGEs to RAGE results in the formation of intracellular ROS [18,118]. Age-adjusted SAF levels in ICU patients were considerably higher than in control participants (*p* < 0.001) in a prospective observational study with 35 ICU patients and a control group (*n* = 231). AGE buildup was not related to illness severity, ICU duration of stay, or death [119]. Higher sRAGE concentrations have been detected in critically ill patients, and they have been linked to future organ failure (e.g., the requirement for renal replacement therapy) and hospital mortality. After controlling for baseline risk variables and disease severity, these relationships remained substantial for circulatory and kidney failure [120,121]. The kidney is both the perpetrator and the target of RAGE signaling in individuals with septic acute kidney damage [19]. The initial rise in sRAGE is most likely due to the inflammatory load and might be due to the expression of the shortened receptor, the local proteolytic environment, or both [121]. Another small study, however, found no elevated blood sRAGE levels in the setting of acute kidney injury [122].

## 9. Transplantation

Because kidney transplantation is intended to restore eGFR, it is expected to reduce circulating AGE concentrations. Despite this, AGEs remain higher than usual and disproportionally high in relation to eGFR, suggesting that additional variables, such as increased oxidative stress, may impact AGE production in the context of kidney transplant recipients [123]. Kidney transplantation is associated with higher levels of long-term, continuing, low-grade inflammation, which is negatively related to circulating CML concentrations via RAGE-mediated trapping of CML in adipose tissue [124]. Elevated circulating AGEs are also a result of increased formation and impaired renal clearance in kidney transplant patients. AGEs activate a number of intracellular pathways that result in an increase in CVD. Table 5 describes the characteristics of human studies investigating the role of AGEs and sRAGE in transplantation [4,99,101,123,124,125,126,127].

A direct association between CML and CEL concentrations and long-term risk of CV mortality was found in a prospective cohort study of 555 stable kidney transplant recipients with a median follow-up of 6.9 years, independent of traditional CV risk factors [124]. These two primary glycation-free adducts in CKD patients drive inflammation, oxidative stress, and endothelial dysfunction [128,129]. The most important independent predictor of circulating CML and CEL concentrations was eGFR. AGE-RAGE-mediated activation of intracellular pathways underpins, at least to a significant extent, the negative connection between AGEs and long-term risk of CV mortality by inducing oxidative stress and the production of endothelial dysfunction indicators [124]. The interaction between AGE and RAGE promotes the production of VCAM-1, which may impact vascular remodeling in transplant vasculopathy. AGEs have been associated with arterial stiffness, increased coronary atherosclerosis, cardiac remodeling, and ventricular dysfunction in CVD [101]. More research is needed to determine whether measuring these AGEs might assist monitoring stable kidney transplant recipients, estimating prognosis, and adapting existing therapy. It is necessary to study if AGE-targeted therapies can provide therapeutic approaches to lower excess CV illness after kidney transplantation and the burden of early CV death in successful kidney transplant patients [124]. Another study of 591 kidney transplant patients found that mycophenolate mofetil, creatinine clearance, BMI, and fasting insulin concentration were all independent predictors of sRAGE. Low sRAGE levels were linked to a 2–3 times increased risk of death, even when adjusting for creatinine clearance [130].

In a study including 630 kidney transplant patients and 41 healthy kidney donors, the median urinary excretion rates of CML and CEL were lower pre-donation but higher post-donation. With the exception of N-G-carboxyethylarginine (CEA), kidney donation resulted in a reduction in the urine excretion rates of all AGEs. Kidney donation may alter the production of methylglyoxal and glyoxal, which are precursors of CEA and N-G-carboxymethylarginine (CMA), respectively. Lower urinary CML and furosine excretion rates were linked to increased all-cause mortality in kidney transplant recipients. Furthermore, reduced urinary furosine excretion rates were associated with increased CV mortality [4]. CML and CEL’s carboxymethyl and carboxyethyl groups raise the affinity constants for RAGE’s V-domain [131]. Urinary furosine excretion rate was inversely related to nephropathy, whereas urinary CML excretion rate was related to the intake of prednisolone [4]. Methylprednisolone has been shown to increase *AGER* gene expression in primary human keratinocytes [132].

After kidney transplantation, AGEs may have a role in the development of CVD and chronic kidney transplant failure [133,134]. SAF revealed a substantial decrease in AGE levels in kidney transplant recipients compared to dialysis patients in a study including 285 kidney transplant recipients, 32 dialysis patients, and 231 healthy control subjects (*p* < 0.0001). However, SAF levels in transplant patients remained significantly higher than in healthy controls (*p* < 0.0001). As a result, kidney transplantation cannot totally repair dialysis patients’ increased AGE tissue levels. After transplantation, there was a negative connection between SAF levels and creatinine clearance (*p* < 0.0001). The existing disparities in kidney function are most likely to explain the differences in fluorescence between control participants, dialysis patients, and transplant recipients. Other factors, in addition to kidney function, contribute to the increased AGE amount following donation. After transplantation, there may be a pool of irreversible AGEs or a de novo generation of AGEs as a result of increased oxidative stress caused by immunosuppressive medicines such as cyclosporin or changes in AGE food consumption [99]. Furthermore, age, smoking, systolic blood pressure, high-sensitive CRP (hsCRP), plasma vitamin C concentrations, pre-transplant dialysis duration, creatinine clearance at baseline, and change in creatinine clearance to baseline 12 months after transplantation were all found to be independently associated with AGE elevation in kidney transplant recipients. Some of these relationships point to AGEs playing a causal role in chronic transplant failure and CVD in kidney transplant patients. Prospective studies with AGE-lowering treatment may help to evaluate the relative impact of AGE accumulation in the development of chronic kidney transplant failure and CVD after transplantation with more certainty [125].

In T1DM patients, simultaneous pancreas–kidney transplantation (SPKT) can restore glycemic control and kidney function, reducing AGE accumulation. Prior to transplantation, plasma pentosidine concentrations in kidney and SPKT candidates were 20 to 35 times higher (*p* < 0.001) than in healthy controls. Following transplantation, plasma pentosidine levels in the kidney (*p* < 0.0001) and SPKT (*p* < 0.005) decreased significantly. In reversing increased plasma pentosidine levels after SPKT, correction of kidney failure is more crucial than the restoration of euglycemia. However, the correction is insufficient, and more than two years after transplantation, plasma levels of pentosidine remained more than thrice higher than in healthy people. Because these individuals seldom have an adequate kidney function, prolonged renal insufficiency may account for the slight but persistent rise in plasma pentosidine concentrations in both kidney and SPKT patients. In the partial correction of pentose-derived glycation of plasma proteins, SPKT has no benefit over kidney transplantation alone [126]. A transitory rise in blood CML levels is found after a SPKT, followed by a drop in CML levels beginning 3 months after transplantation. Mean CML values decreased considerably when comparing CML levels at the beginning to CML levels 12 months after transplantation (*p* = 0.022). Several well-known inflammatory/infectious insults, as well as high dosages of new drugs such as immunosuppressors, might explain the first elevation in these markers after SPKT. Major surgery, indwelling catheters, wound episodes, and urinary, abdominal, or systemic infections may all contribute to an early inflammatory state in SPKT patients [127].

## 10. Conclusions

AGEs may be among the key contributors to the development of kidney diseases, raising oxidative stress in the body through multiple mechanisms and causing an inflammatory response. These processes have a significant impact on the onset and worsening of kidney dysfunction. The observed connections between sRAGE and kidney pathology, as well as associated consequences, may likely be a result of reduced kidney filtration rather than a complication of hyperglycemia. Further study findings will assist in elucidating the function of AGEs and sRAGE in kidney disorders.

sRAGE levels may be affected by numerous molecules in the AGE-RAGE pathway; however, many studies have only examined sRAGE or a combination of sRAGE, CML-AGE, and esRAGE, only capturing a fraction of the AGE-RAGE pathway [60]. The AGE/sRAGE ratio seems to be a better marker to estimate patient’s risk than AGEs or sRAGE alone [9].

Because of their stability in body fluids, the validation of AGEs as biomarkers in kidney diseases would not be a problem. Despite the achievements of the current analytical methods for measuring AGEs and sRAGE, there are still questions about their cost, complexity, and the variability of their results. There is a need for more standardized, noninvasive techniques with simpler applications and improved dependability [15].

The traditional treatment of chronic kidney diseases may alter in the future as novel therapeutic drugs to inhibit AGE-RAGE-mediated pathogenic pathways are developed [135,136]. Another issue will be to prevent AGEs from having receptor-independent effects. The analysis of RAGE polymorphisms to identify variants with protective or detrimental effects on kidney disease has also been proposed, as it would allow a more individualized treatment of CKD patients.

## Figures and Tables

**Table 1:** Potential effects of specific AGEs in development of kidney diseases.

AGE	Potential Working Mechanisms in Kidney Diseases	Ref.
CEL	Induction of endothelial dysfunction and vascular wall inflammatory activation.	[46]
CML	CML activates NF-κB, which in turn increases ZEB2 expression. Increased ZEB2 expression orchestrates podocyte destruction in two ways: (1) ZEB2 reduces E-cadherin expression, allowing podocytes to undergo epithelial-mesenchymal transition and detach from the basement membrane, resulting in a lower podocyte count per glomerulus. (2) ZEB2 also inhibits P-cadherin expression, which is found on SD protein. Reduced P-cadherin expression results from impaired SD function. CML also inhibits nephrin, another important protein in SD, by an unknown mechanism. The loss of E- and P-cadherins, as well as nephrin, results in a drop in podocyte count and proteinuria. CML enhances Notch signaling in podocytes, which contributes to EMT. RAGE-dependent endothelial dysfunction and arterial stiffness induction.	[25,47,48,49,50]
Furosine	Furosine binds to aldose reductase and may harm the kidney by causing lysis of renal cells and the buildup of peroxides in ferroptosis. Furosine causes cell death in cultured human cell lines in a dose-dependent manner.	[51,52,53]
MetSOX	Methionine is a direct ROS target and is highly oxidation-prone, resulting mostly in free and protein-bound MetSOX. Chronic dietary methionine consumption may cause vascular and renal damage, as well as tubular hypertrophy. High plasma methionine and MetSOX concentrations may produce a gradual increase in the GFR, compromising renal function.	[54]
MG-H1	MG-H1 is the AGE with the largest endogenous flow of production in CKD, vastly outnumbering MG of exogenous origin. In CKD, Glo1 is down-regulated, which causes MG buildup. The creation of MG-H1 results in the loss of charge and all electrostatic contacts of arginine residue modification, hence removing functional interactions and activities. Arginine residues are most likely to be found in protein functional domains (20%). The MG modification degrades protein function and is most commonly seen on functionally critical arginine residues. The dicarbonyl proteome refers to proteins that have been changed by MG and associated dicarbonyl metabolites. Protein targets of MG glycation include (1) collagen-4, which is preferentially modified by MG at integrin binding sites, leading to endothelial cell detachment, increased circulating endothelial cells, and vascular damage; (2) mitochondrial proteins, which lead to increased ROS formation; (3) LDL, which induces pro-atherogenic transformation to small, dense, low-density lipoprotein, leading to dyslipidemia; (4) p65 of the NF-kB pathway, which causes increased expression of RAGE, S100A8, S100A12, and HMGB1, as well as increased and chronic vascular inflammation; and (5) apolipoprotein-A1, which causes HDL instability and contributes to dyslipidemia. Dicarbonyl stress promotes vascular renal inflammation as well as renal and muscular fibrosis.	[55]
Pentosidine	Pentosidine is linked to inflammation and oxidative stress. Pentosidine plays an essential role in endothelial dysfunction by reducing levels of two major endothelium-dependent relaxing factors (NO and PGI2) and boosting endothelial production of the powerful vasoconstrictor ET-1. Contribution to immune system dysregulation. Involvement in arterial stiffness.	[1,2]

Abbreviations: AGE: advanced glycation end product; CEL: N^6^-carboxyethyl-l-lysine; CKD: chronic kidney disease; CML: N^6^-carboxymethyl-l-lysine; EMT: epithelial-mesenchymal transition; ET-1: endothelin-1; GFR: glomerular filtration rate; Glo1: glyoxalase 1; HDL: high-density lipoprotein; LDL: low-density lipoprotein; MetSOX: methionine sulfoxide; MG(-H1): methylglyoxal-derived hydroimidazolone; NF-κB: nuclear factor kappa-light-chain-enhancer of activated B cells; NO: nitric oxide; P-cadherin: placental cadherin; PGI2: prostaglandin I2; RAGE: receptor for advanced glycation end products; ROS: reactive oxygen species; SD: slit-diaphragm; ZEB2: zinc finger E-box binding homeobox 2.

**Table 2:** Overview of human studies investigating the role of AGEs and sRAGE in chronic kidney disease.

Study Design	Study Participants	Types of Detection Method	End Point	Key Findings	Ref.
Cohort study	64 older individuals (70% male, median age of 81 years, 63% DM, steady eGFR (27 ± 10 mL/min/1.73 m^2^)	AGEs: fluorescence spectrophotometry sRAGE: ELISA (Human RAGE DuoSet, R&D)	To evaluate the association between the amount in variations in AGEs and sRAGE, and eGFR.	Mean AGEs levels remained nearly the same with rather steady eGFR, although sRAGE isoforms decreased significantly (*p* < 0.0001). AGEs/sRAGE ratios rose (*p* = 0.004), but variations in AGEs and RAGEs isoforms were not associated with changes in eGFR.	[9]
Cohort study	111 advanced CKD patients (CKD stages 3b to 5, not yet on dialysis)	AGEs: fluorescence spectrophotometry sRAGE (Human RAGE DuoSet, R&D)/cRAGE (∆(sRAGE-esRAGE)/esRAGE (B-Bridge International K1009-1): ELISA	To explore the role of AGE, glycated albumin, sRAGE and its different forms, as prognostic factors for mortality.	eGFR correlated negatively with AGE, sRAGE, esRAGE and cRAGE. No differences were observed between diabetic and non-diabetic patients. AGE, esRAGE, and cRAGE/esRAGE were independently associated with all-cause mortality.	[10]
Cohort study	123 ESKD including HD (*n* = 56) and PD patients (*n* = 67)	sRAGE: ELISA (Human RAGE DuoSet, R&D)	To explore whether sRAGE may be a predictor of mortality in ESKD.	A rise in sRAGE levels of 100 pg/mL was linked with a substantial increase in mortality risk of roughly 7%. A positive correlation between sRAGE and BNP was found.	[11]
Cross-sectional study	548 older community-dwelling women (51.6% with eGFR < 60 mL/min/1.73 m^2^)	AGEs (CML): ELISA sRAGE: ELISA (Quantikine human RAGE, R&D)	To characterize the relationship between AGEs and RAGE with CVD mortality.	Increased serum CML and sRAGE were independently linked with lower eGFR and seemed to predict reduced eGFR	[56]
Cross-sectional study	1874 participants	AGEs (CML): ELISA sRAGE (Quantikine human RAGE, R&D)/esRAGE (B-Brigde International): ELISA	To evaluate associations of AGE-CML, sRAGE and esRAGE with demographics, DM, hyperglycemia, cardiometabolic measures, and genetic variants in the *AGER* gene.	DM was not linked with serum AGE-CML, sRAGE, or esRAGE after adjusting for baseline demographic variables and BMI. Black race and *AGER* genetic variations were highly related to lower sRAGE and esRAGE concentrations. eGFR, albuminuria, and CKD risk based on eGFR and albuminuria stages were linked to increased levels of AGE-CML, sRAGE, and esRAGE. Fructosamine and glycated albumin were linked with AGE-CML. AGE-CML and sRAGE were linked to genetic, renal, and inflammatory markers rather than DM.	[57]
Case-control study	88 ESKD patients and 20 healthy controls	AGEs: ELISA (Oxiselect AGE, STA-817, Cell Biolabs) sRAGE: ELISA (Human RAGE DuoSet, R&D)	To investigate the higher increases in serum levels of AGEs compared to increases in sRAGE, the correlation between the levels of AGEs with sRAGE, and the increase in the ratio of AGEs/sRAGE in patients with ESKD.	The AGEs levels were 6.75 times higher in ESKD patients compared to the controls. The rise in AGE levels was 2–3.23 times greater than the increase in sRAGE levels. A negative connection was discovered between sRAGE and the AGEs/sRAGE ratio; the latter might be used as a general biomarker for ESKD.	[58]
Cohort study	1634 HD patients	AGEs: spectrofluorometric and SAF	To investigate the association of serum and tissue AGEs with all-cause and CVD mortality	There was a positive relation of baseline tissue AGE levels with all-cause and CVD mortality, independent of circulating AGEs and other important confounders.	[59]
Case-control study	261 stable HD patients and 100 unrelated Caucasian healthy people	sRAGE: ELISA (Quantikine human RAGE, R&D)	To evaluate sRAGE, inflammatory, and nutritional parameters, and to determine *AGER* polymorphisms.	A negative relationship between residual diuresis and acute-phase reactants (fibrinogen and orosomucoid) was found. sRAGE levels were linked to kidney functions, AGEs, and inflammatory markers, and could be altered genetically. The greatest sRAGE levels were reported in *AGER* gene polymorphisms 429 CC and 2184 GG. sRAGE levels were not linked to mortality.	[60]
Cohort study	199 HD patients	sRAGE: ELISA (Quantikine human RAGE, R&D)	To evaluate the relationship between sRAGE and S100A12 and mortality.	sRAGE was adversely linked with vascular calcification scores but not with inflammatory markers. Plasma sRAGE and S100A12 were not linked with mortality.	[61]
Case-control study	200 CKD stage 5 patients with initiation of dialysis (CKD-G5D) (median age: 56 years, 62% men and median GFR of 6.2 mL/min/1.73 m^2^), 58 HD patients,78 PD patients, 56 CKD stage 3–4 patients and 50 community-based control subjects	sRAGE: ELISA (Quantikine human RAGE, R&D)	To investigate plasma S100A12 and sRAGE, biomarkers of inflammation and nutritional status, and comorbidities and to assess associations between mortality risk and S100A12 or sRAGE.	Plasma concentrations of sRAGE, S100A12, and the ratio S100A12/sRAGE were significantly higher in CKD-G5D, CKD stage 3–4, HD and PD patients. Median level of sRAGE was higher in CKD-G5D patients compared to patients with CKD stage 3–4. sRAGE was adversely associated with GFR, but not with hsCRP, comorbidities, or mortality.	[62]
Case-control study	81 patients (25 patients of CKD stage 1–4, 20 long-term HD patients, 15 PD patients, and 21 healthy age-matched subjects)	sRAGE: ELISA (Quantikine human RAGE, R&D)	To describe the relationship of sRAGE to kidney function and dialysis modalities.	Serum sRAGE levels increased in patients with decreased kidney function, mainly patients with ESKD.	[63]
Cohort study	107 T2DM patients including those on HD	AGEs (CML and pentosidine): ELISA (FSK, Fushimi Pharmaceutical) esRAGE: ELISA (B-Bridge International)	To investigate the role of esRAGE and to determine whether serum esRAGE levels are associated with serum AGEs levels.	In uremia, an elevated sRAGE level may imply an increased RAGE expression within a counter-regulatory mechanism against vascular endothelial injury.	[64]
Case-control study	82 prevalent PD patients (median age: 65 years; 70% men), 190 HD patients (median age: 67 years; 56% men), and 50 control participants (median age: 63 years; 62% men)	sRAGE: ELISA (Quantikine human RAGE, R&D)	To assess associations between mortality risk and concentrations of S100A12 and sRAGE.	Median S100A12, sRAGE, and S100A12/sRAGE were significantly higher than in controls, with S100A12 being 1.9 times higher and median sRAGE being 14% lower in HD patients.	[65]

Details of antibodies ELISA kits: Human RAGE DuoSet R&D: mouse anti-human RAGE capture antibody and biotinylated goat anti-human RAGE detection antibody; B-Bridge International K1009-1: capture anti-RAGE antibody + detection antibody (esRAGE antibody horseradish peroxidase-conjugated); Quantikine human RAGE; R&D: monoclonal capture antibody specific for human RAGE (extracellular domain) + polyclonal detection antibody specific for human RAGE (extracellular domain) conjugated to HRP; CML-ELISA: a CML-specific monoclonal antibody (mouse monoclonal 4G9; Alteon, Ramsey, NJ, USA); Oxiselect AGE, STA-817, Cell Biolabs: anti-AGE antibody and secondary antibody; AGE MyBioSource: a monoclonal antibody specific to AGE (antibody targets conformational epitope rather than linear epitope); sRAGE MyBioSource: capture antibody specific for sRAGE + HRP-conjugated human sRAGE detection antibody; FSK pentosidine ELISA: pentosidine-specific rabbit antibody + HRP-labeled goat anti-rabbit IgG polyclonal antibody; FSK CML ELISA: CML-specific rabbit antibody + HRP-labeled goat anti-rabbit IgG polyclonal antibody. Abbreviations: *AGER*: advanced glycosylation end product-specific receptor; AGEs: advanced glycation end products; BNP: brain natriuretic peptide; CKD: chronic kidney disease; CKD-G5D: CKD stage 5 on dialysis; CML: N^6^-carboxymethyl-l-lysine; cRAGE: cleaved RAGE; CRP: C-reactive protein; CV: cardiovascular; CVD: cardiovascular disease; DM: diabetes mellitus; eGFR: estimated glomerular filtration rate; ELISA: enzyme-linked immunosorbent assay; ESKD: end-stage kidney disease; esRAGE: endogenous secretory RAGE; GFR: glomerular filtration rate; HD: hemodialysis; hsCRP: high-sensitivity CRP; PD: peritoneal dialysis; RAGE: receptor for advanced glycation end products; S100A12: S100 calcium-binding protein A12; SAF: skin autofluorescence; sRAGE: soluble RAGE; T2DM: type 2 diabetes.

**Table 3:** Overview of human studies investigating the role of AGEs and sRAGE in diabetic nephropathy.

Study Design	Study Participants	Types of Detection Method	End Point	Key Findings	Ref.
Cohort study	ACCORD (*n* = 1150) and VADT (*n* = 447), T2DM patients	AGEs: LC-MS	To evaluate the association of a multicomponent AGE panel with decline in kidney function and its utility in predicting RFL.	The data provided further support for a causal role of AGEs in DN independently of glycemic control and suggested utility of the composite AGE panel in the prediction of long-term decline in kidney function.	[6]
Cohort study	ONPN (*n* = 14), NHS (*n* = 110), Pima Indians (*n* = 45), three populations with DM	-	To examine the relationship of methylglyoxal, 3-deoxyglucosone, and oxidative stress levels to DN risk.	Progression of DN was significantly related to elevated dicarbonyl stress and possibly related to oxidative stress in three separate populations, suggesting that these factors play a role in determining individual susceptibility.	[34]
Cohort study	103 subjects with T1DM	AGEs: LC MS-MS	To investigate the relationship between AGEs, oxidation products, and progression of DN.	Fast progressors had considerably higher levels of MG-H1, CEL, and CML than slow progressors. MG-H1 was found to be a substantial independent predictor of rapid progressors. The findings imply that the three main AGEs may be early indications of the advancement of significant DN damage.	[35]
Cohort study	169 American Indians with T2DM and early stage DN (mean eGFR > 90 mL/min, and mean uACR 31 mg/g) from the Gila River Indian Community	AGEs: LC-MS	To examine associations of AGEs with RFL and its structural determinants.	Serum AGEs predicted RFL. AGEs improved prediction of RFL and its major structural correlates.	[36]
Case-control study	Tissue studies: 9 diabetic patients and 18 non-diabetic patients Clinical studies: 20 diabetic patients (6 undergoing HD patients) and 10 non-diabetic patients with ESKD requiring HD, and 8 healthy subjects	AGEs: radioreceptor assay	To elucidate the relation of AGEs to diabetic complications.	AGEs accumulated at a faster-than-normal rate in arteries and the circulation of patients with DM. The increase in circulating AGE peptides paralleled the severity of functional kidney impairment in DN.	[83]
Cohort study	466 participants	AGEs: LC-MS	To assess impact of glycemic control on plasma AGEs and their association with subsequent microvascular disease.	MetSOX was linked to DKD at TP1. A positive relation between fructose-lysine and CML and albuminuria was discovered when age, BMI, DM duration, and mean updated HbA1c were taken into account.	[84]
Case-control study	150 CKD stage 3–5 patients and 64 non-CKD patients	sRAGE: ELISA (Quantikine human RAGE, R&D)	To investigate the effect of glycemic control on the levels or activities of sRAGE in CKD patients.	Glycemic control did not quantitatively alter sRAGE in diabetic CKD patients. The presence of CKD may negate the impact of glycemic management on enzymatic antioxidant activity and sRAGE levels.	[85]
Cross-sectional study	76 CKD-G5D patients (32 HD and 44 PD patients, median age 62.41 years) of which 24 with DM (T1DM: *n* = 2; T2DM: *n* = 22) and 54 without DM	sRAGE: ELISA (Quantikine human RAGE, R&D)	To evaluate the existence of any potential association between AGEs, FGF-23, inflammation, and increased CV risk in CKD-G5D patients and to explore the potential role of sRAGE as a marker of heart failure.	sRAGE levels were higher in DM CKD-G5D patients compared to non-DM CKD-G5D patients. Its elevation may be a possible preventive mechanism against increased risk of CV problems associated with AGEs and inflammation.	[86]
Cohort study	3725 patients (mean eGFR: 82.6 ± 21.3 mL/min/1.73 m^2^, median uACR: 2.2 (0.8–8.8) mg/mmol)	AGEs: SAF	To investigate the association between SAF and progression of kidney disease.	Higher SAF values were associated with progression of kidney disease and with the eGFR slope in T2DM, after adjustment for established risk factors.	[87]

Details of antibodies ELISA kits: Quantikine human RAGE, R&D: monoclonal capture antibody specific for human RAGE (extracellular domain) + polyclonal detection antibody specific for human RAGE (extracellular domain) conjugated to horseradish peroxidase. Abbreviations: ACCORD: Action to Control Cardiovascular Risk in Diabetes; AGEs: advanced glycation end products; BMI: body mass index; CEL: N^6^-carboxyethyl-l-lysine; CKD: chronic kidney disease; CKD-G5D: CKD stage 5 on dialysis; CML: N^6^-carboxymethyl-l-lysine; CV: cardiovascular; DKD: diabetic kidney disease; DM: diabetes mellitus; DN: diabetic nephropathy; eGFR: estimated glomerular filtration rate; ESKD: end-stage kidney disease; FGF-23: fibroblast growth factor 23; HbA1c: hemoglobin A1c; HD: hemodialysis; LC: liquid chromatography; MetSOX: methionine sulfoxide; MG-H1: methylglyoxal-derived hydroimidazolone; MS: mass spectrometry; MS-MS: tandem MS; NHS: Natural History of Diabetic Nephropathy study; ONPN: Overt Nephropathy Progressor/Non-progressor; PD: peritoneal dialysis; RAGE: receptor for AGEs; RFL: renal function loss; SAF: skin autofluorescence; sRAGE: soluble RAGE; T1DM: type 1 diabetes; T2DM: type 2 diabetes; TP: timepoint; uACR: urine albumin-to-creatinine ratio; VADT: Veterans Affairs Diabetes Trial.

**Table 4:** Overview of human studies investigating the role of AGEs and sRAGE in atherosclerosis.

Study Design	Study Participants	Types of Detection Method	End Point	Key Findings	Ref.
Cohort study	200 CKD stage 5 patients (62% men, median of 56 years, and median GFR of 6.2 mL/min/1.73 m^2^) starting on dialysis.	sRAGE: ELISA (Quantikine human RAGE, R&D)	To assess associations between mortality risk and sRAGE after a median follow-up period of 23 months.	sRAGE does not seem to be a valid risk marker in this CKD stage 5 patient population.	[62]
Case-control study	58 HD, and 78 PD patients after 1 year of dialysis, 56 CKD stage 3–4 patients and 50 community-based control subjects	sRAGE: ELISA (Quantikine human RAGE, R&D)	To compare sRAGE levels in different patients.	sRAGE plasma concentrations are markedly elevated in CKD stage 5 patients starting on dialysis, as well as in CKD stage 3–4 patients and prevalent dialysis patients. CKD stage 5 patients had higher median level of sRAGE than the CKD stage 3–4 patients.	[62]
Case-cohort study	151 CKD patients (eGFR < 60 mL/min/1.73 m^2^ and ≥ 25% eGFR decline), 152 ESKD patients, and 1218 healthy patients	sRAGE: ELISA sRAGE: ELISA (Quantikine human RAGE, R&D)	To examine the association between sRAGE and kidney disease.	High sRAGE levels were associated with the development of CKD and ESKD risk, but not after adjustment for kidney function at baseline.	[97]
Cross-sectional study	Ambulatory adult patients (*n* = 51) with CKD stages 1 through 5 and without DM	AGEs: ELISA (CML and MG) RAGE (mRNA): SYBR^TM^ Green real-time PCR assay	To examine the association of AGE accumulation with cellular RAGE expression and endothelial dysfunction as well as the mechanisms of this association in CKD.	RAGE mRNA expression rises in the presence of high amounts of circulating AGEs, resulting in up-regulation of RAGE synthesis.	[98]
Case-control study	285 transplant recipients (mean age: 52 years), 32 dialysis patients (mean age: 56 years), 231 normal healthy subjects (mean age: 51 years)	AGEs: SAF	To evaluate AGE levels.	Kidney transplantation does not fully correct increased AGE levels found in dialysis patients. Increased AGE levels in kidney transplant recipients cannot be explained by the differences in kidney function alone.	[99]
Cohort study	70 patients in the AAA group (55 men and 15 women, mean age: 70.25 years), 20 patients in the AIOD group (14 men and 6 women, mean age: 63.78 years), and 85 CKD patients (CKD stage 3–4) and 35 HD patients	AGEs: ELISA (Oxiselect AGE, STA-817, Cell biolabs) sRAGE: ELISA (RayBio)	To identify the relationship between AGEs, sRAGE, and AGE/sRAGE in selected atherosclerosis diseases: AAA, AIOD, and CKD.	The CKD patients had substantially greater quantities of AGEs and sRAGE than the patients with AAA and AIOD. sRAGE was considerably higher in CKD stage 5 patients than in CKD stage 3–4 patients. Significantly higher AGEs/sRAGE ratio was also seen in HD patients compared to those with AAA and AIOD.	[100]
Case-control study	6 patients with ESKD and DM, 8 patients with ESKD and without DM, and 8 patients without ESKD and without DM	AGEs: immunohistochemistry	To study the accumulation of AGEs and apolipoprotein B in the human aortas in diabetic and non-diabetic subjects with ESKD.	It is suggested that impaired AGE clearance may cause the increased accumulation of AGEs in the aortic wall of subjects with ESKD, thus resulting in rapid progression of atherosclerosis.	[101]
Case-control study	142 CKD patients (average eGFR of 32 mL/min/1.73 m^2^) and 49 healthy control individuals matched for age and gender	sRAGE: ELISA (Human RAGE DuoSet, R&D)	To determine the relationship between plasma sRAGE and carotid atherosclerosis.	Plasma sRAGE was significantly higher in patients with CKD than in the control cohort. In CKD patients, significant inverse relationships were found for sRAGE to IMT and plaque number. The slopes of IMT and plaque number to sRAGE were significantly steeper in patients with CKD. For predicting atherosclerotic plaques in patients with CKD, a significant interaction was found between sRAGE and smoking.	[102]
Case-control study	142 patients with average eGFR of 32 mL/min/1.73 m^2^ and 49 healthy control individuals matched for age and gender	sRAGE: ELISA (Human RAGE DuoSet, R&D)	To study the relationship between plasma sRAGE with LVH in CKD patients.	sRAGE is an inverse marker of LVH in CKD patients.	[103]

Details of antibodies ELISA kits: Human RAGE DuoSet, R&D: mouse anti-human RAGE capture antibody and biotinylated goat anti-human RAGE detection antibody; Quantikine human RAGE, R&D: monoclonal capture antibody specific for human RAGE (extracellular domain) + polyclonal detection antibody specific for human RAGE (extracellular domain) conjugated to horseradish peroxidase; Oxiselect AGE, STA-817, Cell Biolabs: anti-AGE antibody and secondary antibody; CML ELISA: 4G9 mAb; Alteon, Northvale, NJ; MG ELISA: MG3D11 mAb; RayBio: capture antibody specific for human RAGE + biotinylated antihuman RAGE antibody. Abbreviations: AAA: abdominal aortic aneurysm; AGEs: advanced glycation end products; AIOD: aortoiliac occlusive disease; CKD: chronic kidney disease; CML: N^6^-carboxymethyl-l-lysine; DM: diabetes mellitus; eGFR: estimated glomerular filtration rate; ELISA: enzyme-linked immunosorbent assay; ESKD: end-stage kidney disease; GFR: glomerular filtration rate; HD: hemodialysis; IMT: intima–media thickness; LVH: left ventricular hypertrophy; MG: methylglyoxal; mRNA: messenger ribonucleic acid; PD: peritoneal dialysis; PCR: polymerase chain reaction; RAGE: receptor for AGEs; SAF: skin autofluorescence; sRAGE: soluble RAGE; SYBR: Synergy Brands.

**Table 5:** Overview of human studies investigating the role of AGEs in transplantation.

Study Design	Study Participants	Types of Detection Method	End Point	Key Findings	Ref.
Case-control study	630 kidney transplant patients and 41 healthy kidney donors	AGEs: GC-MS	To investigate the associations of urinary AGEs with mortality.	Median urinary excretion rates of CML and CEL were lower pre-donation but higher post-donation. Lower urinary CML and furosine excretion rates were linked to increased all-cause mortality in kidney transplant recipients. Reduced urinary furosine excretion rates were linked to increased CV mortality. Urinary furosine excretion rate was inversely related to nephropathy, whereas urinary CML excretion rate was related to prednisolone.	[4]
Case-control study	285 transplant recipients (mean age: 52 years), 32 dialysis patients (mean age: 56 years), and 231 normal control subjects (mean age: 51 years)	AGEs: SAF	To evaluate AGE levels in the skin.	SAF revealed a substantial decrease in AGE levels in kidney transplant recipients compared to dialysis patients. However, fluorescence levels in transplant patients remained significantly higher than in healthy controls. A negative correlation between fluorescence levels and creatinine clearance was found after transplantation.	[99]
Case-control study	6 patients with ESKD and DM, 8 patients with ESKD and without DM, and 8 patients without ESKD and without DM	AGEs: immunohistochemistry	To study the accumulation of AGEs and apolipoprotein B in the human aortas of diabetic and non-diabetic subjects with ESKD.	It is suggested that impaired AGE clearance may cause the increased accumulation of AGEs in the aortic wall of subjects with ESKD, resulting in rapid progression of atherosclerosis.	[101]
Case-control study	28 healthy controls, 11 conservatively treated children with CRI, all dialyzed children with CRI (HD: *n* = 8 or PD: *n* = 18), and 9 children after kidney transplantation	AGEs: fluorimetry and ELISA (CML)	To investigate the pattern of AGE accumulation in children/adolescents with CRI and on renal replacement therapy by dialysis and after transplantation.	Enhanced fluorescent AGEs and CML levels were found in children/adolescents with CRI and on dialysis. Successful kidney transplantation decreased but did not normalize AGE levels. This probably because of an impaired kidney function with enhanced oxidative stress.	[123]
Cohort study	555 stable kidney transplant recipients (mean age: 51 years, 56% men)	AGEs: UPLC-MS-MS	To investigate the association between circulating AGEs and long-term risk of CV mortality.	CML and CEL concentrations were directly associated with CV mortality, independent of traditional CV risk factors.	[124]
Cohort study	285 consecutive kidney transplant recipients (57% male, mean age: 50 years)	AGEs: SAF	To investigate which factors are associated with tissue AGE accumulation in kidney transplant recipients.	Age, smoking, systolic blood pressure, hsCRP, plasma vitamin C concentrations, pre-transplant dialysis duration, creatinine clearance at baseline, and change in creatinine clearance at baseline 12 months after transplantation were all found to be independently associated with AGE elevation in kidney transplant recipients.	[125]
Case-control study	128 patients with kidney or kidney-pancreas transplantation (diabetic recipients of a kidney-pancreas transplant: *n* = 38; diabetic recipients of a kidney-only transplant: *n* = 44; non-diabetic recipients of a kidney transplant: *n* = 46) and 26 healthy volunteers	AGEs: HPLC-fluorescence detection	To determine the effects of correcting hyperglycemia and/or kidney failure on the accumulation of pentosidine.	Prior to transplantation, plasma pentosidine concentrations in kidneys and kidney-pancreas transplant candidates were 20–35 times higher than in healthy controls. Following transplantation, plasma pentosidine levels decreased significantly. More than two years after transplantation, plasma levels of pentosidine remained more than thrice higher than in healthy people.	[126]
Cohort study	20 T1DM patients undergoing pancreas–kidney transplantation	AGEs: ELISA AGE (Oxiselect AGE, STA-817, Cell Biolabs) and CML (Oxiselect CML, STA-816, Cell Biolabs)	To study AGE evolution after pancreas–kidney transplantation.	A transitory rise in CML levels is found after transplantation, followed by a drop in CML levels beginning 3 months after transplantation. Mean CML values decreased considerably when comparing CML levels at the beginning to CML levels 12 months after transplantation.	[127]

Details on antibodies ELISA kits: CML-ELISA: monoclonal antibody against CML; Oxiselect AGE, STA-817, Cell Biolabs: anti-AGE antibody and secondary antibody; Oxiselect CML STA-816, Cell Biolabs: anti-CML monoclonal antibody + horseradish peroxidase-conjugated secondary antibody Abbreviations: AGEs: advanced glycation end products; BMI: body mass index; CEL: N^6^-carboxyethyl-l-lysine; CML: N^6^-carboxymethyl-l-lysine; CRI: chronic renal insufficiency; CV: cardiovascular; DM: diabetes mellitus; ELISA: enzyme-linked immunosorbent assay; ESKD: end-stage kidney disease; GC: gas chromatography; HD: hemodialysis; HPLC: high-performance liquid chromatography; hsCRP: high-sensitivity CRP; MS: mass spectrometry; MS-MS: tandem MS; PD: peritoneal dialysis; RAGE: receptor for AGEs; SAF: skin autofluorescence; T1DM: type 1 diabetes; UPLC: ultra-performance liquid chromatography.

## Data Availability

Not applicable.
